# Mapping neurogenic dysphagia diagnostics in Germany: accessibility, implementation practices, and barriers to swallowing endoscopy

**DOI:** 10.1186/s42466-026-00473-9

**Published:** 2026-03-18

**Authors:** Julia Gelenar Marae, Lars Masanneck, Marc Pawlitzki, Cinja Huber, Jule Hofacker, Sriramya Lapa, Anne Jung, Paul Muhle, Sonja Suntrup-Krueger, Tobias Warnecke, Sven G. Meuth, Rainer Dziewas, Bendix Labeit

**Affiliations:** 1 Department of Neurology, Medical Faculty, University Hospital Düsseldorf, Moorenstraße 5, 40225 Düsseldorf, Germany; 2 Department of Neurology, Goethe University Frankfurt, University Hospital Frankfurt, Frankfurt/Main, Germany; 3 Department of Neurology, University Hospital Münster, Albert-Schweitzer- Campus 1, A1, 48149 Münster, Germany; 4 Department of Neurology and Neurorehabilitation, Klinikum Osnabrueck, Am Finkenhügel 1, 49076 Osnabrueck, Germany

**Keywords:** Dysphagia, Fiberoptic Endoscopic Evaluation of Swallowing (FEES), Healthcare Accessibility, Outpatient Services, Neurogenic Dysphagia

## Abstract

**Background:**

Flexible Endoscopic Evaluation of Swallowing (FEES) is a core diagnostic tool in neurogenic dysphagia. Despite a structured national training curriculum in Germany, little is known about current implementation practices and outpatient accessibility. This study mapped nationwide FEES practice patterns, workforce structures, and geographic access, and identified barriers to comprehensive service provision.

**Methods:**

A nationwide web-based survey was conducted among certified FEES instructors in Germany. The survey assessed provider characteristics, implementation practices, diagnostic–therapeutic integration, and perceived systemic barriers. In parallel, institutional websites were screened for publicly advertised outpatient FEES services and geocoded for travel-time isochrone analyses.

**Results:**

Eighty-two instructors completed the anonymous survey (response rate 52.6%). FEES was predominantly delivered in acute hospitals (74%) and rehabilitation clinics (46%) and embedded within interdisciplinary workflows. Outpatient availability remained limited (54%), with most institutions lacking statutory reimbursement pathways; additionally, many existing outpatient services were not visible to patients online. Geospatial analysis revealed substantial regional disparities, particularly in rural and eastern regions, with only a minority of residents able to reach a publicly identifiable outpatient FEES provider within 30 min. Procedural experience varied widely, and higher monthly FEES volumes were associated with shorter examination duration (*r* = − 0.27, *p* = 0.020).

**Conclusions:**

FEES is well implemented in German inpatient dysphagia care but structurally underdeveloped in the outpatient sector. Insufficient reimbursement, workforce shortages, and lack of transparency significantly impede equitable access. Strengthening outpatient infrastructure, establishing sustainable reimbursement mechanisms, and improving national visibility of services are critical steps toward ensuring continuity of dysphagia care.

**Supplementary Information:**

The online version contains supplementary material available at 10.1186/s42466-026-00473-9.

## Introduction

Dysphagia, or swallowing impairment, is a common and clinically significant manifestation across numerous neurological conditions [1]. It affects a large proportion of patients with stroke [2], Parkinson’s disease [3], other neurodegenerative disorders [4], neuromuscular diseases such as myositis [5] or myasthenia gravis [6], and neuroimmunological conditions including multiple sclerosis and related disorders [7, 8]. In addition, age-related swallowing alterations, termed presbyphagia [9], may contribute to its development, as approximately half of otherwise healthy adults over the age of 70 years show some degree of swallowing alteration [10]. Untreated or inadequately managed dysphagia can result in malnutrition [11], dehydration [12], aspiration pneumonia [11], problems with medication intake [13], and reduced quality of life [14], thereby contributing substantially to morbidity, mortality, and increased healthcare costs [11, 15]. Accurate and timely assessment of swallowing function is therefore essential for effective diagnosis and targeted therapy.

Because swallowing cannot be directly observed from the outside, and key symptoms such as aspiration may remain silent and thus go unnoticed by patients or during bedside clinical assessment, instrumental diagnostics that visualize swallowing are considered the gold standard for diagnosis. In this context, both Videofluoroscopic Swallowing Studies (VFSS) and Flexible Endoscopic Evaluation of Swallowing (FEES) can reliably detect swallowing pathologies and characterize dysphagia phenotypes and are therefore regarded as complementary gold-standard procedures [16]. Each method, however, has distinct methodological advantages and limitations that influence its clinical use. VFSS enables visualization of the oral, pharyngeal, and esophageal phases of swallowing but requires access to a radiological unit and exposes patients to ionizing radiation. In contrast, FEES primarily visualizes the pharyngeal phase of swallowing but offers several unique advantages: It is easily accessible, allows direct visualization of secretion management, enables assessment of laryngeal sensation [17, 18], can be performed at the bedside or in the patient’s home as a mobile procedure, and permits real-time evaluation of therapeutic maneuvers. Owing to these advantages, FEES has become a widely established and versatile tool for the instrumental assessment of dysphagia [2, 19], and is now recommended in numerous clinical guidelines [20–22]. In Germany, FEES is supported by a standardized training and certification program jointly developed by the German Society of Neurology (DGN), the German Stroke Society (DSG) and the German Society of Geriatrics (DGG) [23]. At the European level, this curriculum has been adopted by the European Society for Swallowing Disorders (ESSD) [24]. The program promotes structured education, high procedural standards, and defines a hierarchical certification pathway culminating in the designation of FEES Instructor as the highest level of competence.

Despite its clinical importance and widespread establishment, access to FEES, particularly in the outpatient sector, appears inconsistent. While FEES is routinely available in hospitals and rehabilitation clinics, patients in ambulatory care often face substantial barriers to instrumental swallowing diagnostics. Consequently, many individuals with chronic dysphagia after hospital discharge or those presenting with dysphagia as a leading symptom may not receive adequate assessment or follow-up. To date, however, systematic data on the current provision, implementation practices, and accessibility of FEES services in Germany are lacking.

The present study aimed to address this knowledge gap by conducting a nationwide survey among certified FEES instructors to map the current state of FEES implementation in Germany. Specifically, we sought to 1) characterize the professional backgrounds and institutional settings of FEES providers, 2), examine implementation practices and the integration of FEES into dysphagia management, 3), identify perceived barriers to service provision, particularly in the outpatient sector, and 4) analyze the geographic accessibility of outpatient FEES services from a patient perspective. Together, these data provide the first comprehensive overview of FEES practice in Germany and identify structural and systemic factors that may inform future healthcare planning and policy development.

## Methods

### Survey design and recruitment

We conducted a nationwide survey to map the provision, implementation practices, and barriers to FEES in Germany, as well as the accessibility of outpatient FEES services. The survey was implemented in LimeSurvey (LimeSurvey GmbH, Hamburg, Germany) and was accessible online from April 12 to July 2, 2024.

All FEES instructors listed in the official trainer directory of the DGN were invited to participate (listing as of February 2024). Of 181 listed instructors, 156 could be contacted by e-mail; the remainder had no available contact information, were retired, or had no identifiable institutional affiliation. To prevent duplicate participation, personalized access codes were generated, and non-responders received up to three reminders. The study design was approved by the Ethics Committee of the University Hospital Düsseldorf (2022–2121).

### Survey structure

The survey consisted of a public and an anonymous component, which were implemented as two independent surveys with separate links but distributed within the same invitation email. The public survey included four items requesting the respondent’s name, institutional address, availability of outpatient FEES services, and the year of initial FEES implementation at the institution. The anonymous survey included 39 items, primarily single- and multiple-choice questions supplemented by free-text fields for numerical estimates and comments. Completion of either survey component was voluntary and not mandatory for participation in the other.

Items addressed respondents’ demographic and professional background, institutional characteristics, patient populations, experience and implementation practices regarding FEES (including annual number and average duration of procedures), integration of therapeutic interventions, and perceived accessibility and barriers to FEES in inpatient and outpatient settings. Full questionnaires are provided as Supplementary Material 1.

### Web-based screening of outpatient FEES services

To assess the publicly visible outpatient FEES landscape, we conducted a systematic web-based screening of all institutions affiliated with certified FEES instructors listed in the official trainer directory of the DGN as of November 2025 using a predefined protocol.

The search strategy included:

1. Structured navigation of institutional websites, covering departments of neurology, otolaryngology, geriatrics, speech-language pathology, and outpatient clinics where available
2. Keyword-based searches using combinations of “FEES”, “Flexible Endoscopic Evaluation of Swallowing”, “Dysphagie” “Schlucksprechstunde”, “Dysphagieambulanz”, and “Schluckdiagnostik”; and
3. Identification of explicit statements indicating the provision of outpatient dysphagia services.

All findings were independently reviewed by a second rater, and discrepancies were resolved through discussion. Institutions without publicly accessible information on outpatient dysphagia services were classified as *“not publicly visible outpatient service*,*”* even if such services may exist internally. Outpatient-only institutions (e.g., private practices) operated by a certified FEES instructor and explicitly offering dysphagia services were classified as outpatient FEES providers.

All identified outpatient locations were geocoded and included in the subsequent travel-time isochrone analyses.

### Geospatial analysis

Driving-time-based isochrones were generated to assess geographic accessibility of outpatient FEES services. Isochrones represent geographic contours of equal travel time by road and provide a more realistic measure of healthcare accessibility than straight-line distance. Isochrones were calculated for car travel times of 15, 30, 45, and 60 min, with 60 min selected as the upper threshold for the primary analysis.

Geospatial analyses were performed using Python 3.13.3 (Python Software Foundation, Delaware, USA) with the packages pandas (v2.3.3), geopandas (v1.1.1), rasterio (v1.4.3), and shapely (v2.1.2). Geocoding was conducted using geopy (v2.4.1) with the Nominatim geocoder and OpenStreetMap (OSM) data. Isochrones were calculated using the openrouteservice Python client (v2.3.3) with a locally hosted instance of OpenRouteService (Docker container image v9.3.0). Isochrones for each center were aggregated through union operations to determine coverage areas for each travel-time category.

The resulting geographic layers were applied as masks to the Global Human Settlement Population Grid R2023A (GHS-POP) [25, 26] to estimate the population covered in 2025. Visualizations were generated using matplotlib (v3.10.7).

### Statistical analysis

Survey data were exported to IBM SPSS Statistics (v29.0.2.0, IBM Corp., Armonk, NY, USA) for analysis. Descriptive statistics were applied: Nominal variables are reported as absolute and relative frequencies (n, %), and continuous variables as means (± SD) or medians with interquartile ranges (IQR), as appropriate. To assess the relationship between procedural workload and examination efficiency, we calculated Pearson’s correlation coefficient between the self-reported average number of FEES procedures performed per month and the average duration of a FEES examination.

### Use of artificial intelligence

ChatGPT-5.1 and ChatGPT 5.2 (OpenAI) was used to assist with language editing. All content was reviewed and approved by the authors.

## Results

A total of 156 FEES instructors were invited to participate in the anonymous main survey, of whom 82 provided complete responses (response rate = 52.6%). In addition, 58 respondents (37.2%) completed the public survey, which primarily collected institutional information and the availability of outpatient FEES services. Unless otherwise stated, the results presented below refer to the anonymous main survey (*n* = 82).

### Providers of FEES: Personnel, Teams and Institutions

Respondents represented a multiprofessional workforce of physicians, speech-language pathologists (SLPs), and academic SLPs. As summarized in Table 1, the cohort was predominantly mid-career (mean age 48.9 years) with a balanced gender distribution.

Most participants worked in acute hospitals (74%) or rehabilitation clinics (46%), whereas outpatient practices remained uncommon (6%). FEES examinations were predominantly conducted in interdisciplinary physician–SLP teams (55%), while 39% were SLP-only procedures; exclusively physician-led FEES was rare.

Teams varied in size and structure. Institutions reported a median of six dysphagia team members and approximately four staff members independently qualified to perform FEES.

**Table 1:** Characteristics of FEES Providers: Demographic and professional characteristics of survey respondents, including age, gender distribution, professional background, institutional settings, and typical team structures involved in performing and interpreting FEES

Variable	Total Cohort
*Demographics*	
Men, n (%)	37 (45.1%)
Women, n (%)	45 (54.9%)
Age, mean ± SD	48.9 ± 9.6
*Professional group*,* n (%)*	
Physician	37 (45.1%)
SLPs	26 (31.7%)
Academic SLPs	15 (18.3%)
Other	4 (4.9%)
*Qualification (non-physician)*,* n (%)*	
Vocational training	22 (26.8%)
Bachelor	5 (6.1%)
Master	8 (9.8%)
Doctorate	4 (4.9%)
*Medical specialty (physicians)*,* n (%)*	
Neurology	29 (35.4%)
Geriatrics	11 (13.4%)
Internal medicine	5 (6.1%)
ENT	2 (2.4%)
Phoniatrics	1 (1.2%)
*Institution*,* n (%)*	
Acute hospital	61 (74.4%)
Rehabilitation clinic	38 (46.3%)
Self-employed practice	5 (6.1%)
Employed practice	1 (1.2%)
*Institutional field*,* n (%)*	
Neurology	61 (74.4%)
Geriatrics	9 (11.0%)
SLP-institution	5 (6.1%)
ENT	1 (1.2%)
Other	6 (7.3%)
Team size (mean ± SD)	5.87 ± 3.84
Persons trained to perform FEES, mean ± SD	4.19 ± 2.40
Persons routinely performing FEES, mean ± SD	3.99 ± 2.53
*FEES performed by*,* n (%)*	
Physicians only	2 (2.4%)
Physicians and SLPs	45 (54.9%)
SLPs only	32 (39.0%)
Other constellation	3 (3.7%)
*FEES interpreted by*,* n (%)*	
Physicians only	6 (7.3%)
Interdisciplinary	33 (40.2%)
SLPs only	43 (52.4%)

FEES = Flexible Endoscopic Evaluation of Swallowing; SLP = Speech-Language Pathologist; SD = Standard Deviation

### Examined patient groups

The most frequently assessed patient group was stroke (83%), followed by multifactorial geriatric patients (17%), Parkinson’s disease (13%), and oropharyngeal cancer (2%). When asked to name the second most frequently assessed group, respondents most often cited Parkinson’s disease (39%) and geriatric dysphagia (33%).

Almost all institutions reported performing FEES in patients with tracheostomy (99%), showing that tracheostomy management is an established part of routine FEES services.

### Experience and implementation practices in FEES

Respondents reported substantial variability in procedural experience. The total number of FEES performed ranged widely from a few hundred to over 10,000 (Table 2). Monthly FEES activity also differed markedly between institutions, indicating diverse structural capacities and case volumes across the country.

**Fig. 1:**
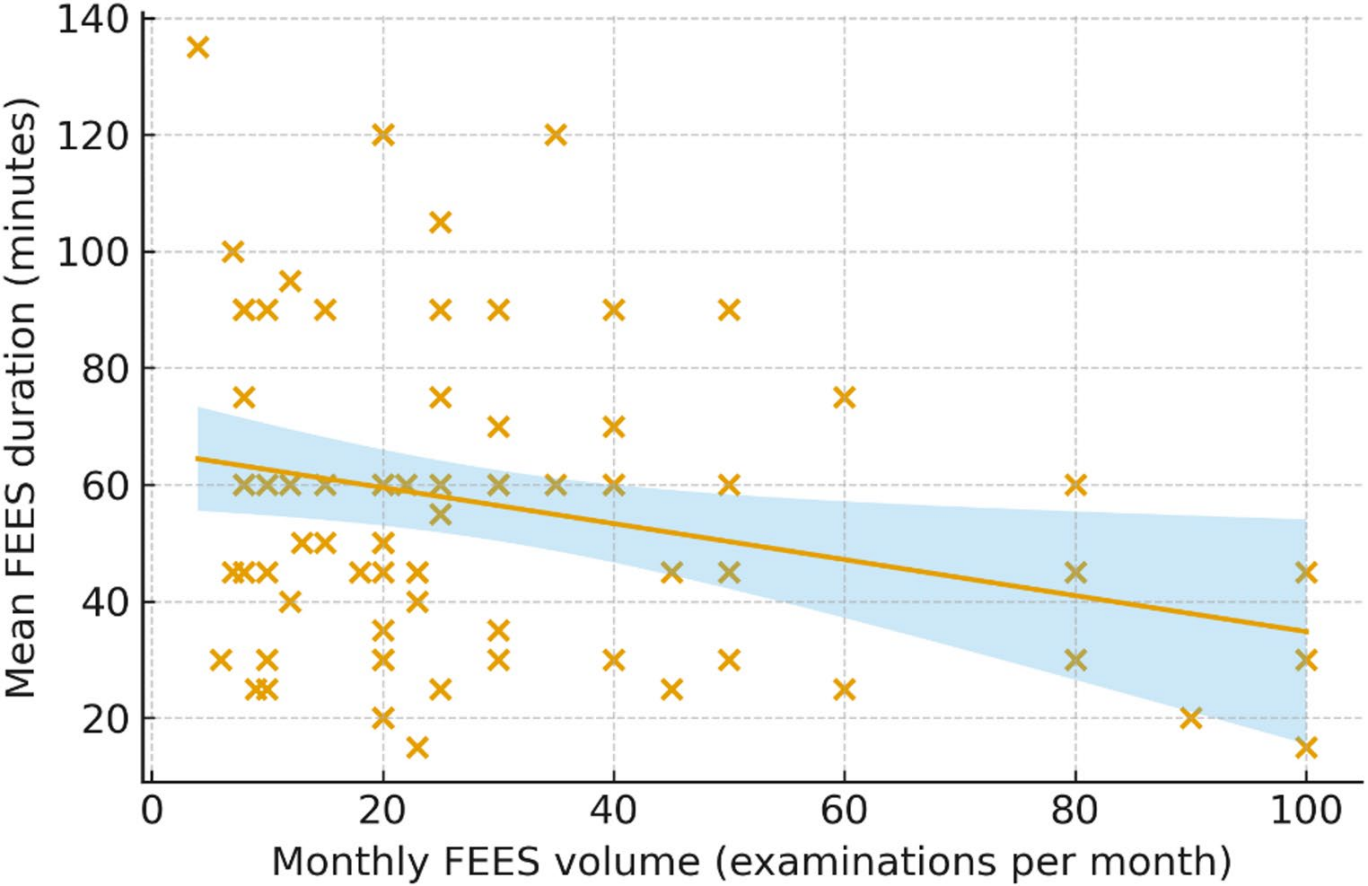
Relationship between monthly FEES volume and mean procedure duration. Scatter plot showing the association between the average number of FEES examinations performed per month and the mean duration of a complete FEES procedure (including preparation, examination, and reporting). Each point represents one certified FEES instructor. A linear regression line with 95% confidence interval (shaded area) is shown. Higher monthly FEES volumes were associated with shorter examination duration (*r* = − 0.27, *p* = 0.020), indicating greater procedural efficiency in higher-volume settings. FEES = Flexible Endoscopic Evaluation of Swallowing

A complete FEES procedure (including preparation, examination, and reporting) required on average 56 min, but durations ranged considerably. Notably, there was a significant negative correlation between procedural volume and FEES duration (*r* = − 0.265, *p* = 0.020). Figure 1 illustrates the relation between monthly FEES-volume and average duration of a FEES in a scatter plot.

**Table 2:** Procedural Experience and Implementation: Self-reported procedural experience and implementation metrics among FEES instructors, including total FEES performed and interpreted, monthly examination volumes in inpatient and outpatient settings, and the average duration of a complete FEES procedure

Variable	Mean ± SD	Range
Total FEES performed	1,827 ± 2,036	210–10,000
Total FEES interpreted	2,046 ± 2,353	210–15,000
FEES per month (inpatient)	29.26 ± 23.64	4–100
FEES per month (outpatient)	8.21 ± 10.86	0–45
Duration of FEES (minutes)	56.2 ± 27.8	5–135

FEES = Flexible Endoscopic Evaluation of Swallowing; SD = Standard Deviation

### Integration of diagnosis and therapy in FEES

Nearly all institutions (94%) used FEES to evaluate therapeutic interventions such as compensatory strategies or texture modifications (Table 3). Despite this, only 29% offered regular outpatient dysphagia therapy, revealing a structural gap between diagnosis and continuity of care, as behavioral treatment is often provided by external therapists who were not involved in the FEES examination.

Respondents estimated that FEES findings were adequately incorporated into approximately half (51%) of externally delivered therapy episodes. Limited therapist expertise, technical barriers to transferring videos or reports, and time constraints were the most frequently cited reasons for inadequate consideration of FEES-findings.

Access to additional instrumental diagnostics was heterogeneous: 54% reported onsite VFSS and 26% high-resolution manometry (HRM), while 40% had no further instrumental assessments available.

**Table 3:** Integration of FEES findings into dysphagia therapy across institutions, including availability of outpatient dysphagia therapy, estimated weekly therapy volumes, and the proportion of therapy episodes adequately incorporating FEES results

Variable	*n* (%) / Mean ± SD
FEES used to evaluate dysphagia therapy	77 (93.9%)
Outpatient dysphagia therapy offered	24 (29.3%)
Patients with outpatient therapy (per week)	11.6 ± 15.2
Estimation of percentage with adequate consideration of FEES in therapy	51.2 ± 30.4
Estimation of percentage with inadequate consideration of FEES in therapy	9.9 ± 7.9
Estimation of percentage with no consideration of FEES in therapy	6.9 ± 9.6
*Reasons for insufficient consideration of FEES in therapy*	
Lack of competence	33 (40.2%)
Technical barriers	22 (26.8%)
Time constraints	14 (17.1%)
*Additional diagnostics (internal)*	
VFSS	44 (53.7%)
HRM	21 (25.6%)
None	33 (40.2%)
*Additional diagnostics (external)*	
VFSS	17 (20.7%)
HRM	13 (15.9%)
None	18 (22.0%)

FEES = Flexible Endoscopic Evaluation of Swallowing; HRM = High Resolution Manometry; SD = Standard Deviation; VFSS: Videofluoroscopic Swallowing Study

### Accessibility and barriers to outpatient FEES services

Outpatient FEES was available in 54% of institutions; however, only 22% operated a dedicated dysphagia clinic and 6% offered mobile FEES (Table 4). Billing options were highly restricted and most commonly available only for privately insured or self-paying patients. A uniform reimbursement pathway through statutory health insurance was absent.

The supplementary web-based search identified 59 affiliations associated with FEES instructors who publicly advertised outpatient FEES services. By contrast, 34 respondents in the public survey reported offering outpatient FEES, but for 14 of these institutions no publicly accessible information could be found, suggesting that such services may in some cases exist but remain invisible and therefore difficult to access for patients.

**Table 4:** Availability of outpatient FEES services across institutions, types of outpatient structures (including dedicated dysphagia clinics and mobile services), reimbursement options, and the most frequently reported barriers to establishing or expanding outpatient FEES capacity

Variable	*n* (%)
*FEES in outpatient service*	
Available	44 (53.7%)
With dysphagia clinic	18 (22.0%)
Mobile FEES	5 (6.1%)
*Billing option*	
Private insurance	23 (28.0%)
Self-paying	20 (24.4%)
University outpatient lump sum	7 (8.5%)
*Barriers to FEES in outpatient service*	
Lack of reimbursement	29 (35.4%)
Lack of staff capacity	21 (25.6%)
Lack of institutional support	17 (20.7%)
Lack of competence	2 (2.4%)
Lack of equipment	4 (4.9%)
Lack of interest by institution	7 (8.5%)
No demand	1 (1.2%)
Estimated full demand coverage (inpatient)	50 (61.0%)
Estimated full demand coverage (outpatient)	18 (22.0%)

FEES = Flexible Endoscopic Evaluation of Swallowing

**Fig. 2:**
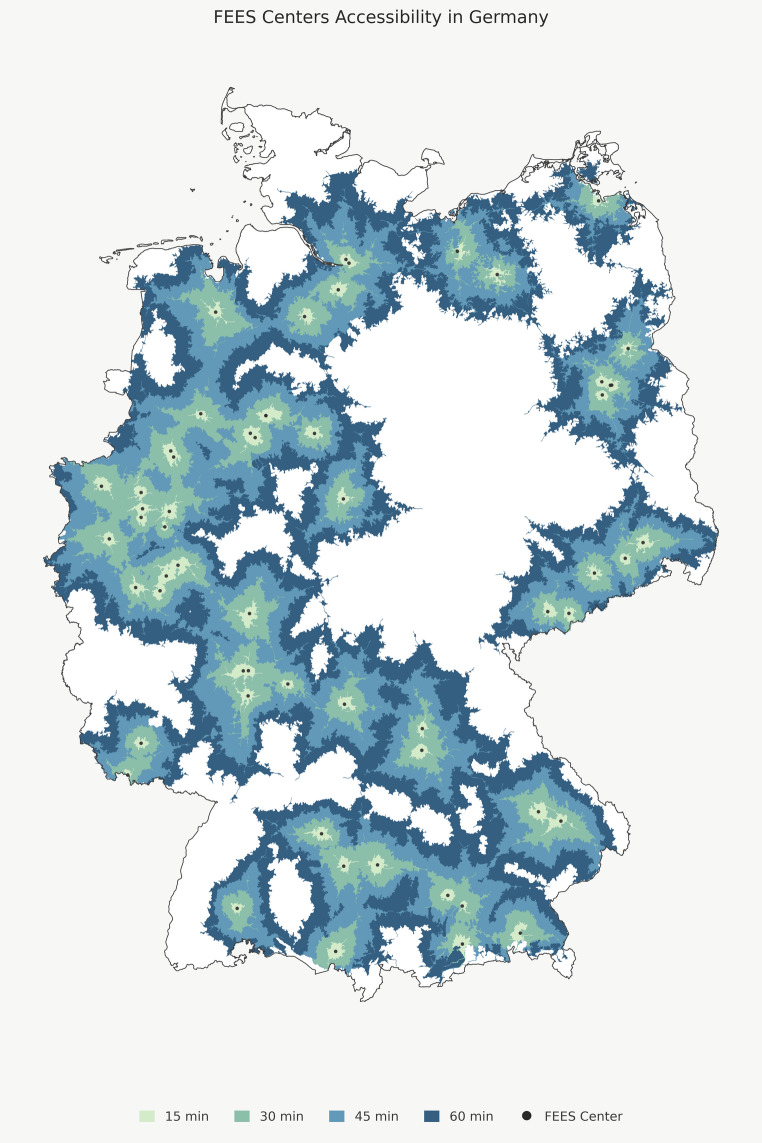
Geographic accessibility of publicly advertised outpatient FEES services in Germany. Map visualizing travel-time isochrones around identified outpatient FEES centers (black points). Isochrones represent estimated car travel times: 15-minute catchment areas (blue), 30-minute (yellow), 45-minute (orange), and 60-minute (red). The figure illustrates the uneven spatial distribution of outpatient FEES availability, with dense coverage in metropolitan regions and limited accessibility across large rural areas

Isochrone analyses revealed pronounced regional disparities: 10.5% of the population could reach an outpatient FEES provider within 15 min, 38.4% within 30 min, 61.1% within 45 min, and 78.1% within 60 min. Dense clusters occurred mainly in western and southern Germany, whereas extensive rural areas particularly in eastern Germany had limited coverage even within a 60-minute travel radius (Fig. 2). Substantial disparities between federal states were evident: Densely populated regions such as North Rhine-Westphalia demonstrated high accessibility. In contrast, large rural states including Thuringia and Saxony-Anhalt showed markedly lower coverage, with fewer than 10% of residents within a 60-minute travel radius. Supplementary Material 2 provides a detailed description of the national isochrone analysis, while Supplementary Material 3 presents the corresponding state level results.

## Discussion

This nationwide survey provides a comprehensive overview of the current implementation, practice patterns, accessibility, and barriers of FEES in Germany. The findings demonstrate that FEES is well established as a routine diagnostic tool in neurological and geriatric dysphagia care, particularly within hospital based and rehabilitative settings. At the same time, major gaps persist regarding outpatient service provision, integration into therapeutic pathways, and reimbursement structures. Together, these results highlight the success of the German FEES training program while underscoring the need for system level improvements to ensure equitable access across care settings.

### Multiprofessional practice and institutional characteristics

Consistent with the interdisciplinary nature of the German FEES curriculum [23], FEES is conducted by a wide range of professionals, including neurologists, geriatricians, and SLPs. The predominance of interdisciplinary examination teams reflects a strong collaborative culture. In the context of this survey, ‘FEES interpretation’ primarily refers to the functional endoscopic assessment of swallowing and to therapy- and nutrition-related recommendations, rather than to medical decision-making. Integration of these functional findings into overall patient management relies on close collaboration with the treating physician, particularly in patients with neurogenic dysphagia, where neurological expertise is required to contextualize findings within the broader clinical framework.

### Experience, scalability, and quality of FEES implementation

The survey revealed wide variability in procedural experience among certified FEES instructors. Lifetime examination numbers ranged from a few hundred to more than ten thousand procedures. Importantly, higher monthly FEES volume was associated with shorter examination duration, a finding that likely reflects a combination of factors, including greater procedural routine and more standardized workflows, as well as differences in patient populations, clinical indications, and diagnostic complexity across institutions. In particular, FEES performed in acute stroke settings with standardized indications and established workflows [2] differs fundamentally from more complex diagnostic evaluations, such as those in patients with neuromuscular disorders [5, 6, 27], parkinsonian syndromes [28, 29], long-standing unexplained dysphagia [6, 30], or multifactorial geriatric conditions [9], which typically require more extensive assessment and interpretation. Similar associations between procedural experience and shorter procedure times have been described in other medical fields and have been interpreted as reflecting greater efficiency and, in some contexts, higher quality of care [31, 32]. However, in the absence of adjustment for patient population and diagnostic complexity, procedural duration in the present study cannot be interpreted as a surrogate for workflow efficiency and quality of care. Nevertheless, from a health services perspective, high-volume centers may function as regional reference points for training, quality assurance, and clinical expertise. Defining minimum recommended examination numbers and other quality criteria could help standardize proficiency levels and promote high quality FEES practice nationwide. Future studies will be required to determine whether such structural factors translate into measurable improvements in workflow efficiency and quality of care.

### Integration of FEES into dysphagia therapy

Nearly universal use of FEES to evaluate therapeutic interventions confirms its dual role as both a diagnostic and therapy evaluation tool in dysphagia management. However, only about half of respondents felt that their FEES findings were consistently integrated into subsequent therapy. Barriers included limited therapist expertise, difficulties transferring videos and reports, and time constraints in outpatient settings. Improved technical infrastructure for video sharing, standardized reporting, and integrated electronic records together with clearer regulatory frameworks particularly with regard to data protection may help ensure that instrumental findings are more effectively translated into individualized therapy.

### Outpatient access and structural barriers

One of the most striking findings of this study is the limited availability of outpatient FEES services. This aligns with international observations indicating that access to instrumental dysphagia diagnostics in long-term care and post-acute settings is often inadequate [33]. Limited outpatient access is particularly relevant from a clinical standpoint because many patients with dysphagia as leading symptom such as in myositis [30], myasthenia gravis [6], or amyotrophic lateral sclerosis [34] first seek care in the outpatient sector, often before an underlying neurological condition has been diagnosed. Without accessible outpatient FEES, early diagnostic clarification and timely referral may be delayed.

The lack of structured follow up after acute hospitalization further complicates care. In patients with acute stroke, recovery of oral intake and PEG dependence often extends beyond the inpatient period. For instance, among patients with percutaneous endoscopic gastrostomy (PEG) feeding, about 30% do not regain sufficient oral intake within 30 days [35]. Without outpatient FEES, clinicians may be unable to adjust nutritional strategies in a timely manner, for example to discontinue PEG feeding once swallowing improves or to initiate PEG placement early enough to prevent clinically relevant weight loss in progressive neurological diseases. The lack of outpatient dysphagia assessment options may also contribute to the broader absence of standardized long term dysphagia pathways described in guideline syntheses on post stroke dysphagia, which has been identified as a major gap in post stroke dysphagia care [36]. Decannulation decisions in tracheostomized patients also often rely on repeated instrumental assessments [37], yet decannulation is predominantly performed in acute hospitals and rarely after discharge [38]. Thus, for many patients, the acute inpatient stay effectively represents the last realistic opportunity for decannulation [38]. Improving outpatient access to FEES may therefore help increase decannulation rates by enabling safe reassessment beyond the acute phase. Enhanced outpatient FEES availability may thus support earlier detection of neurological disorders presenting with dysphagia as a leading symptom, more timely modification of oral intake, better PEG management, structured post acute follow up, and safer and more appropriate decannulation pathways.

Despite these potential benefits, financing remains a major barrier to outpatient FEES. Reimbursement is often restricted to privately insured or self paying patients, with no clear pathway for coverage within statutory health insurance. This creates inequitable access to an essential diagnostic procedure and raises significant ethical concerns.

### Geographic accessibility and regional disparities

Geospatial analyses showed pronounced regional inequalities in FEES availability. Services are concentrated in densely populated regions such as North Rhine Westphalia, Lower Saxony, Hesse, and Bavaria, while many rural and eastern regions such as Thuringia and Saxony-Anhalt lack accessible providers within reasonable travel times.

Importantly, geographic proximity alone does not necessarily translate into effective access to care. Isochrone-based analyses reflect travel-time distance but do not capture critical determinants of real-world accessibility, including outpatient capacity, waiting times, staffing constraints, or institutional differences in reimbursement, for example between university and non-university providers. Consequently, effective access to outpatient FEES may be overestimated even in regions with apparent geographic coverage. In addition, car-based travel times likely represent an optimistic scenario for many patients with neurogenic dysphagia, as older and frail individuals frequently depend on caregivers or institutional transport services, which may substantially limit real-world accessibility despite nominal proximity. Beyond these methodological considerations, the observed spatial disparities reflect broader challenges in rural healthcare provision and highlight the need for regionally coordinated dysphagia networks. Outreach models such as mobile FEES services could help reduce these inequalities [33] and, in addition to addressing geographic access barriers, may also be particularly valuable for patients who are immobile [39]. The web-based assessment also revealed that many institutions offering outpatient FEES do not advertise these services online, limiting visibility for patients and referring clinicians. Publicly accessible and regularly updated registries of certified FEES providers with outpatient services would therefore improve navigation and referral.

### Implications for practice and policy

These findings indicate a need for structural reforms in dysphagia diagnostics and care. Standardized reimbursement codes for outpatient FEES within statutory health insurance are essential to reduce inequities. Integrating FEES into interdisciplinary networks involving SLPs, neurology, gastroenterology, and otolaryngology may strengthen continuity of care. The observed variability in procedural experience emphasizes the value of high-volume centers, which should be supported through defined quality criteria and minimum case numbers.

Experience from stroke care demonstrates the potential impact of structured service models. The establishment of stroke units with defined quality criteria and dedicated reimbursement led to substantial improvements in treatment and reductions in mortality [40]. In a similar manner, the creation of certified dysphagia centers with clear quality requirements, the capacity for outpatient follow up, and appropriate reimbursement pathways may improve dysphagia care and reduce dysphagia related complications. Enhanced technical infrastructure for video exchange, standardized communication pathways between diagnostic and therapeutic teams, and the reduction of legal barriers such as data protection constraints may further increase the clinical impact of instrumental swallowing assessments.

### Limitations

This study has several limitations. First, participation in the nationwide survey was voluntary and may be subject to non-response bias; institutions with well-established FEES programs might therefore be overrepresented. Second, all survey data were self-reported and are inherently prone to recall and reporting bias, particularly regarding procedural experience and workflow parameters. Third, the web-based screening of outpatient FEES services relied solely on publicly accessible online information. As a result, outpatient services that exist but are not explicitly advertised are not included, leading to potential underestimation of true outpatient availability. Fourth, the analysis was limited to certified DGN-FEES instructors, and outpatient providers without formal certification were not captured. Finally, the generalizability of the findings is restricted to the German healthcare context and may not directly translate to other national systems with different reimbursement structures or workforce configurations.

## Conclusion

FEES is well integrated into inpatient dysphagia diagnostics in Germany and is performed by highly trained interdisciplinary teams. However, outpatient accessibility remains limited due to structural, financial, and geographic barriers. Addressing reimbursement deficits, improving communication and technical infrastructure, and expanding regional accessibility are essential steps toward improving diagnostic equity and continuity of care for patients with dysphagia.

## Supplementary Information

Supplementary Material 1

Supplementary Material 2

Supplementary Material 3

## Data Availability

The datasets generated are available from the corresponding author on reasonable request.

## Abbreviations

DGG: German Society of Geriatrics
DGN: German Society of Neurology
ESSD: European Society for Swallowing Disorders
FEES: Flexible Endoscopic Evaluation of Swallowing
GHS-POP: Global Human Settlement Population Grid
HRM: High-Resolution Manometry
IQR: Interquartile Range
OSM: OpenStreetMap
PEG: Percutaneous Endoscopic Gastrostomy
SD: Standard Deviation
SLP: Speech-Language Pathologist
VFSS: Videofluoroscopic Swallowing Study
