# Supplementary material for: Mapping neurogenic dysphagia diagnostics in Germany: accessibility, implementation practices, and barriers to swallowing endoscopy
Source: Neurol Res Pract. 2026 Mar 18;8(1):17. doi: 10.1186/s42466-026-00473-9 (PMC13001358; doi:10.1186/s42466-026-00473-9)
Supplement: Supplementary file 1 — Supplementary Material 1 [file 42466_2026_473_MOESM1_ESM.docx]

**Supplementary Material 1: Umfrage zur Dysphagiediagnostik in Deutschland (Survey on Dysphagia Assessment in Germany)**

1. **Öffentlicher Teil (Public-Response Section)**

**Wie lautet Ihr Vor- und Zuname?**• Freitext

**Gibt es in Ihrer Einrichtung die Möglichkeit einer ambulanten FEES-Diagnostik?**
Bitte wählen Sie eine der folgenden Antworten:
• Nein, FEES wird nur bei stationären Patienten durchgeführt
• Ja, FEES wird bei Ambulanzpatienten durchgeführt, es gibt aber keine spezielle Dysphagieambulanz in der sich Dysphagiepatienten vorstellen können
• Ja, es gibt eine Dysphagieambulanz in der FEES durchgeführt wird
• Ja, wir sind eine rein ambulante Einrichtung

**Falls es in Ihrer Einrichtung die Möglichkeit einer ambulanten FEES-Diagnostik gibt, wie lautet die Adresse der Einrichtung?**
• Freitext

**In welchem Jahr wurde die FEES in Ihrer Einrichtung etabliert?**
• Freitext

1. **Anonymer Teil (Anonymized-Response Section)**

**Was ist Ihr Geschlecht?**
Bitte wählen Sie eine der folgenden Antworten:
• Männlich
• Weiblich
• Divers

**Wie alt sind Sie?**
• Freitext

**Wie viele FEES haben Sie insgesamt ca. durchgeführt?**
Bitte die Anzahl schätzen.
• Freitext

**Wie viele FEES haben Sie insgesamt ca. befundet?**
Bitte die Anzahl schätzen.
• Freitext

**Wie lange dauert die Durchführung einer FEES-Untersuchung im Durchschnitt bei Ihnen in der Einrichtung inklusive Vor- und Nachbereitung und Befundung?**
Bitte in Minuten schätzen.
• Freitext

**Welcher Berufsgruppe gehören Sie an?**
Bitte wählen Sie eine der folgenden Antworten:
• Arzt/Ärztin
• Logopäde/Logopädin
• Akademischer Sprachtherapeut/akademische Sprachtherapeutin
• Sonstiges

**Im Falle von LogopädInnen bzw. SprachtherapeutInnen, was ist Ihr Berufsabschluss?**
(Mehrfachnennung möglich)
Wählen Sie alle zutreffenden Optionen:
• Ausbildungsberuf
• Bachelor
• Master
• Promotion
• Sonstiges:

**Im Falle von ÄrztInnen, was ist Ihr Fachgebiet?**
(Mehrfachnennung möglich)
Wählen Sie alle zutreffenden Optionen:
• Neurologie
• Geriatrie
• Innere Medizin
• HNO
• Phoniatrie
• Sonstiges:

**In was für einer Einrichtung führen Sie die FEES-Untersuchung durch?**
(Mehrfachnennung möglich)
Wählen Sie alle zutreffenden Optionen:
• Akutkrankenhaus
• Rehabilitationsklinik
• Selbstständig in niedergelassener Praxis
• Angestellt in niedergelassener Praxis
• Sonstiges:

**Was für einem Fachbereich lässt sich Ihre Einrichtung am ehesten zuordnen?**
Bitte wählen Sie eine der folgenden Antworten:
• Neurologie
• Hals-Nasen-Ohrenheilkunde
• Phoniatrie
• Geriatrie
• Gastroenterologie
• Logopädie/Sprachtherapie
• Sonstiges:

**Was ist Ihrer Einschätzung nach die häufigste Hauptdiagnose bei PatientInnen, bei denen eine FEES durchgeführt wird?**
Wählen Sie alle zutreffenden Optionen:
• Schlaganfall
• Parkinson-Syndrome
• Multifaktoriell betroffener geriatrischer PatientIn
• Oropharynx-Karzinom
• Sonstiges:

**Was ist Ihrer Einschätzung nach die zweithäufigste Hauptdiagnose bei PatientInnen, bei denen eine FEES durchgeführt wird?**
Wählen Sie alle zutreffenden Optionen:
• Schlaganfall
• Parkinson-Syndrome
• Multifaktoriell betroffener geriatrischer PatientIn
• Oropharynx-Karzinom
• Sonstiges:

**Sind bei Ihnen schon mal PatientInnen mit Trachealkanülen mittels FEES untersucht worden?**
Bitte wählen Sie eine der folgenden Antworten:
• Ja
• Nein

**Falls bei Ihnen PatientInnen mit Trachealkanülen mittels FEES untersucht wurden, wie häufig kommt das im Jahr ca. vor?**
• Freitext

**In welcher Berufsgruppenkonstellation wird die FEES durchgeführt?**
Bitte wählen Sie eine der folgenden Antworten:
• Durch ÄrztInnen
• In einem interdisziplinären Team aus ÄrztInnen und LogopädInnen/SprachtherapeutInnen
• Durch LogopädInnen/SprachtherapeutInnen
• Sonstiges:

**In welcher Berufsgruppenkonstellation wird die FEES befundet und interpretiert?**
Bitte wählen Sie eine der folgenden Antworten:
• Federführend durch ÄrztInnen
• In einem interdisziplinären Team aus ÄrztInnen und LogopädInnen/SprachtherapeutInnen
• Federführend durch LogopädInnen/SprachtherapeutInnen

**Wie groß ist Ihr dysphageologisches Team insgesamt (Anzahl der Vollzeitstellen)?**
• Freitext

**Wie viele Personen des dysphageologischen Teams sind in der Lage FEES durchzuführen und zu befunden?**
• Freitext

**Wie viele Personen des dysphageologischen Teams sind im klinischen Alltag typischerweise mit der Durchführung und Befundung von FEES betraut?**
• Freitext

**Gibt es in Ihrer Einrichtung die Möglichkeit einer ambulanten FEES-Diagnostik?**
Bitte wählen Sie eine der folgenden Antworten:
• Nein, FEES wird nur bei stationären PatientInnen durchgeführt
• Ja, FEES wird bei AmbulanzpatientInnen durchgeführt, es gibt aber keine spezielle Dysphagieambulanz in der sich Dysphagiepatienten vorstellen können
• Ja, es gibt eine Dysphagieambulanz in der FEES durchgeführt wird
• Ja, wir sind eine rein ambulante Einrichtung
• Ja, wir haben die Möglichkeit einer mobilen FEES, die vor Ort bei PatientInnen durchgeführt werden kann
• Sonstiges:

**Falls es in Ihrer Einrichtung die Möglichkeit einer ambulanten FEES-Diagnostik gibt, wie wird diese abgerechnet?**
(Mehrfachnennung möglich)
Wählen Sie alle zutreffenden Optionen:
• SelbstzahlerInnen
• Abrechnung über die private Krankenversicherung
• Über eine Hochschulambulanzpauschale
• Sonstiges:

**Falls es in Ihrer Einrichtung keine Möglichkeit zur ambulanten FEES-Diagnostik gibt, was sind hierfür aus Ihrer Sicht die Gründe?**
(Mehrfachnennung möglich)
Wählen Sie alle zutreffenden Optionen:
• Kein Bedarf
• Fehlende personelle Kapazität
• Fehlende personelle Kompetenz
• Fehlende instrumentelle Kapazität
• Hürden bei der Abrechnung
• Nicht gewünscht durch die Einrichtung
• Fehlende Unterstützung beim Aufbau der Strukturen durch die Einrichtung
• Sonstiges:

**Falls Sie in einem Akutkrankenhaus oder einer Rehabilitationsklinik tätig sind, wie viele FEES-Untersuchungen führen Sie in Ihrer Einrichtung im Schnitt pro Monat im stationären Setting durch?**
• Freitext

**Können Sie in Ihrer Einrichtung den geschätzten Bedarf an stationärer FEES-Diagnostik abdecken?**
Bitte wählen Sie eine der folgenden Antworten:
• Ja, hiermit können die notwendigen dysphageologischen Fragestellungen beantwortet werden
• Nein, es sollten weitere Patienten untersucht werden, aber dies ist aus Kapazitätsgründen nicht möglich
• Keine Antwort

**Falls Sie nicht den geschätzten Bedarf an stationärer Dysphagiediagnostik abdecken können, wie viel Prozent des Bedarfs decken Sie damit schätzungsweise ab?**
Bitte in Prozent schätzen von 0%–100%.
In dieses Feld dürfen nur Zahlen eingegeben werden.
• Freitext

**Falls in Ihrer Einrichtung ambulante FEES durchgeführt wird, wie viele ambulante FEES-Untersuchungen führen Sie im Schnitt pro Monat insgesamt durch?**
In dieses Feld dürfen nur Zahlen eingegeben werden.
• Freitext

**Ist damit der Bedarf an ambulanter FEES-Diagnostik in Ihrer Einrichtung bzw. in Ihrem Einzugsgebiet gedeckt?**
Bitte wählen Sie eine der folgenden Antworten:
• Ja, alle Patienten mit Terminwunsch können innerhalb einer Wartezeit von ca. 3 Wochen bedient werden
• Nein, es können nicht alle Terminwünsche bedient werden bzw. es kommt zu langen Wartezeiten
• Keine Antwort

**Falls der Bedarf an ambulanter FEES-Diagnostik nicht vollständig abgedeckt ist, wie viel Prozent des Bedarfs decken Sie schätzungsweise ab?**
Bitte in Prozent schätzen von 0%–100%.
In dieses Feld dürfen nur Zahlen eingegeben werden.
• Freitext

**Wird in Ihrer Einrichtung Dysphagietherapie (Konsistenzmodifikation, logopädische Übungen etc.) mittels FEES untersucht/evaluiert?**
Bitte wählen Sie eine der folgenden Antworten:
• Ja, wir untersuchen die Wirksamkeit der Dysphagietherapie mittels FEES
• Nein, die Beurteilung der Dysphagietherapie ist bei uns nicht Bestandteil der FEES-Diagnostik
• Keine Antwort

**Wird bei Ihnen in der Einrichtung ambulant regelmäßige Dysphagietherapie (z.B. logopädische Übungen) angeboten?**
Bitte wählen Sie eine der folgenden Antworten:
• Ja, wir haben PatientInnen die regelmäßige ambulante Dysphagietherapie erhalten
• Nein, ambulante regelmäßige Dysphagietherapie wird bei uns nicht angeboten

**Falls bei Ihnen in der Einrichtung ambulante regelmäßige Dysphagietherapie angeboten wird, bei wie vielen PatientInnen die Woche führen Sie schätzungsweise regelmäßige Dysphagietherapie durch?**
In dieses Feld dürfen nur Zahlen eingegeben werden.
• Freitext

**Falls bei Ihnen in der Einrichtung ambulante regelmäßige Dysphagietherapie angeboten wird, wie viel Prozent des Bedarfs wird nach Ihrer Einschätzung dadurch abgedeckt?**
Bitte in Prozent schätzen von 0%–100%.
In dieses Feld dürfen nur Zahlen eingegeben werden.
• Freitext

**Wie erfolgt die regelmäßige Dysphagietherapie bei Ihren PatientInnen, wenn diese außerhalb Ihrer Einrichtung stattfindet?**
Bitte die jeweiligen Prozentzahlen schätzen (alle Optionen müssen beziffert werden, Gesamtwert von 100% muss erreicht werden).
Nur Zahlen dürfen in diese Felder eingegeben werden.

• Bei wie viel Prozent gar nicht, da dies der Patient nicht wünscht?
• Freitext

• Bei wie viel Prozent gar nicht, aufgrund mangelnder Verfügbarkeit?
• Freitext

• Bei wie viel Prozent durch einen anderen logopädischen Kollegen/eine logopädische Kollegin. Meine FEES-Befunde werden hierbei aus meiner Sicht adäquat berücksichtigt.
• Freitext

• Bei wie viel Prozent durch einen anderen logopädischen Kollegen/eine logopädische Kollegin. Meine FEES-Befunde werden hierbei aus meiner Sicht nicht adäquat berücksichtigt.
• Freitext

• Bei wie viel Prozent durch einen anderen logopädischen Kollegen/eine logopädische Kollegin. Meine FEES-Befunde werden hierbei aus meiner Sicht überhaupt nicht berücksichtigt.
• Freitext

**Im Falle der Ansicht, dass die FEES-Befunde durch andere KollegInnen nicht adäquat berücksichtigt werden, was ist Ihrer Ansicht nach hierfür eine entscheidende Ursache?**
(Mehrfachnennung möglich)
Wählen Sie alle zutreffenden Optionen:
• Mangelnde Kompetenz und Schulung der Kollegen bei der Interpretation der FEES-Befunde
• Mangelnde Zeit der KollegInnen, die FEES-Befunde zu berücksichtigen
• Mangelndes Interesse der KollegInnen an der Interpretation der FEES-Befunde
• Technische Hürden wie das Fehlen von datenschutzkonformen und praktikablen Videoweitergabemöglichkeiten
• Sonstiges:

**Wenn die regelmäßige Dysphagietherapie durch andere KollegInnen durchgeführt wird, wie oft werden Sie von diesen nach dem schriftlichen FEES-Befund gefragt bzw. wie oft wird dieser durch PatientInnen weitergegeben?**
Bitte in Prozent schätzen von 0%–100%.
In dieses Feld dürfen nur Zahlen eingegeben werden.
• Freitext

**Wenn die regelmäßige Dysphagietherapie durch andere KollegInnen durchgeführt wird, wie oft werden Sie von diesen nach dem FEES-Video gefragt bzw. wie häufig wird das FEES-Video durch PatientInnen weitergegeben?**
Bitte in Prozent schätzen von 0%–100%.
In dieses Feld dürfen nur Zahlen eingegeben werden.
• Freitext

**Welche weitere instrumentelle Diagnostik ist noch bei Ihnen in der Einrichtung verfügbar?**
(Mehrfachnennung möglich)
Wählen Sie alle zutreffenden Optionen:
• Videofluoroskopie (VFSS)
• High-Resolution Manometrie (HRM)
• Keine
• Sonstiges:

**Falls keine sonstige instrumentelle Diagnostik in der eigenen Einrichtung zur Verfügung steht, zu welcher sonstigen instrumentellen Diagnostik haben Sie über externe Zuweisungen Zugang?**
(Mehrfachnennung möglich)
Wählen Sie alle zutreffenden Optionen:
• Videofluoroskopie (VFSS)
• High-Resolution Manometrie (HRM)
• Keine
• Sonstiges:

**Gibt es sonst noch etwas, was Sie uns sagen möchten?**
• Freitext
