# Supplementary figures and images for: Mapping neurogenic dysphagia diagnostics in Germany: accessibility, implementation practices, and barriers to swallowing endoscopy
Source: Neurol Res Pract. 2026 Mar 18;8(1):17. doi: 10.1186/s42466-026-00473-9 (PMC13001358; doi:10.1186/s42466-026-00473-9)

**Supplementary Materials 2: Geospatial Analysis - National Coverage**


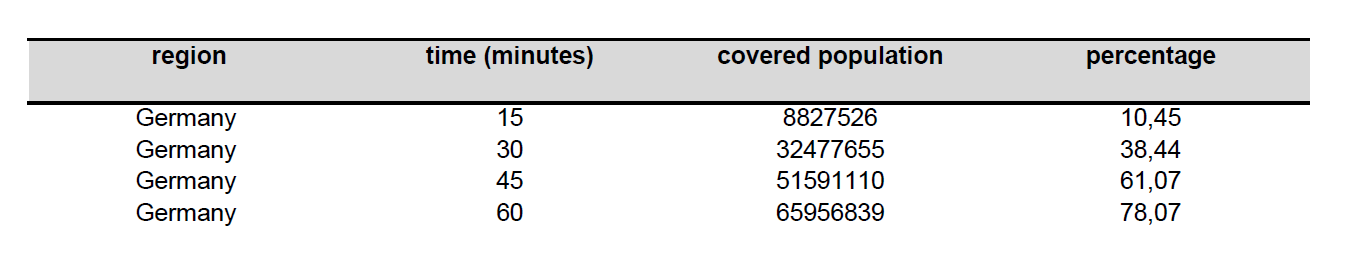

Supplement: Supplementary file 2 — Supplementary Material 2 [file 42466_2026_473_MOESM2_ESM.docx]

**Supplementary Materials 3: Geospatial Analysis - State Coverage**


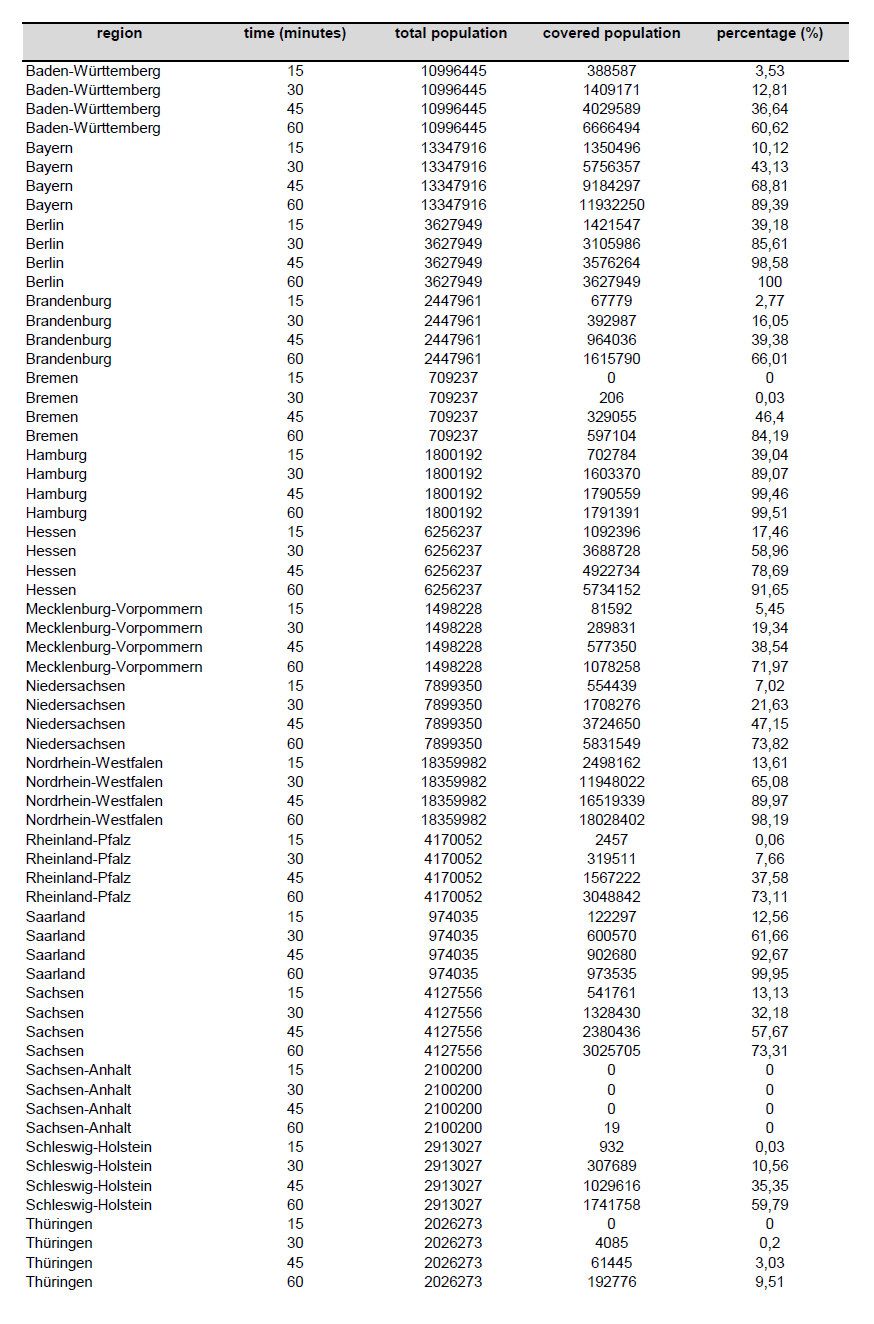

Supplement: Supplementary file 3 — Supplementary Material 3 [file 42466_2026_473_MOESM3_ESM.docx]
